# Defining rules of CD8^+^ T cell expansion against pre-erythrocytic *Plasmodium* antigens in sporozoite-immunized mice

**DOI:** 10.1186/s12936-016-1295-5

**Published:** 2016-04-26

**Authors:** Zachary P. Billman, Arnold Kas, Brad C. Stone, Sean C. Murphy

**Affiliations:** Department of Laboratory Medicine, University of Washington, Seattle, WA USA; Department of Microbiology, University of Washington, Seattle, WA USA; Center for Emerging and Re-emerging Infectious Diseases, University of Washington, Seattle, WA USA

**Keywords:** Malaria, *Plasmodium*, CD8 T cell, Heterologous, Cross-species, Secondary expansion, Late-arresting, RAS, GAP

## Abstract

**Background:**

Whole *Plasmodium* sporozoites serve as both experimental tools and potentially as deployable vaccines in the fight against malaria infection. Live sporozoites infect hepatocytes and induce a diverse repertoire of CD8^+^ T cell responses, some of which are capable of killing *Plasmodium*-infected hepatocytes. Previous studies in *Plasmodium yoelii*-immunized BALB/c mice showed that some CD8^+^ T cell responses expanded with repeated parasite exposure, whereas other responses did not.

**Results:**

Here, similar outcomes were observed using known *Plasmodium berghei* epitopes in C57BL/6 mice. With the exception of the response to PbTRAP, IFNγ-producing T cell responses to most studied antigens, such as PbGAP50, failed to re-expand in mice immunized with two doses of irradiated *P. berghei* sporozoites. In an effort to boost secondary CD8^+^ T cell responses, heterologous cross-species immunizations were performed. Alignment of *P. yoelii* 17XNL and *P. berghei* ANKA proteins revealed that >60 % of the amino acids in syntenic orthologous proteins are continuously homologous in fragments ≥8-amino acids long, suggesting that cross-species immunization could potentially trigger responses to a large number of common Class I epitopes. Heterologous immunization resulted in a larger liver burden than homologous immunization. Amongst seven tested antigen-specific responses, only CSP- and TRAP-specific CD8^+^ T cell responses were expanded by secondary homologous sporozoite immunization and only those to the L3 ribosomal protein and S20 could be re-expanded by heterologous immunization. In general, heterologous late-arresting, genetically attenuated sporozoites were better at secondarily expanding L3-specific responses than were irradiated sporozoites. GAP50 and several other antigens shared between *P. berghei* and *P. yoelii* induced a large number of IFNγ-positive T cells during primary immunization, yet these responses could not be re-expanded by either homologous or heterologous secondary immunization.

**Conclusions:**

These studies highlight how responses to different sporozoite antigens can markedly differ in recall following repeated sporozoite vaccinations. Cross-species immunization broadens the secondary response to sporozoites and may represent a novel strategy for candidate antigen discovery.

**Electronic supplementary material:**

The online version of this article (doi:10.1186/s12936-016-1295-5) contains supplementary material, which is available to authorized users.

## Background

Pre-clinical and clinical vaccine studies have demonstrated that whole *Plasmodium* sporozoites can induce sterile protection against infectious challenge [1–5]. Sporozoite formulations include radiation-attenuated sporozoites (RAS) [3, 6], genetically attenuated parasites (GAP) [7] or wild-type (WT) sporozoites administered under anti-malarial drug prophylaxis [2, 8]. Such approaches induce protective antibodies and T cells with IFNγ-producing cytotoxic T lymphocytes (CTL), which are particularly important for protection during the liver stage [9]. *Plasmodium* are complex eukaryotic pathogens that express thousands of different proteins throughout their lifecycle [10]. Until recently, this enormous array of proteins made it difficult to study antigen-specific immune responses on a large scale. Consequently, relatively little is known about the repertoire of T cell responses and to some extent antibody responses that target pre-erythrocytic stage antigens.

The most well-studied antigen is the circumsporozoite protein (CSP) [11, 12]. CSP encounters antigen-processing cells after being shed from the sporozoite surface [13] and alternatively after transport into the hepatocyte cytoplasm in sporozoite-invaded hepatocyte [14]. CSP induces protective Class I-dependent CTL responses [14–16] and can also induce CD4^+^ T cell responses [17, 18] and major histocompatibility complex (MHC) Class II-dependent IgG responses [19]. While CSP-specific cells can induce protection when present at extremely high frequencies through experimental manipulations [12, 16, 18, 20, 21], such frequencies are not commonly achieved following sporozoite exposures. The CSP-based RTS,S vaccine in humans does not trigger strong CTL responses [22, 23] and instead seems to rely on antibodies and CD4^+^ T cell responses [24] to achieve partial protection [25]. Moreover, non-CSP antigens are increasingly appreciated as potential vaccine candidates since mice can be protected against challenge even in the absence of CSP-specific immunity [15, 26–29]. In addition to CSP, thrombospondin-related adhesive protein (TRAP, also called sporozoite surface protein 2 or SSP2) can induce CD8 + T cells [30] and TRAP-specific CD8 T cells can kill infected liver cells [31]. Like CSP, TRAP is also shed from the sporozoite surface, a process required for gliding motility and sporozoite infectivity [32]. These two proteins have been the focus of most pre-clinical and clinical studies of pre-erythrocytic antigens. While a handful of new antigens have been recently identified [33], the remaining antigens targeted by humoral and cellular immune responses are not well understood.

Minigene library screening was recently employed in an effort to identify novel pre-erythrocytic antigens [34] and identified the L3 ribosomal protein as a target of the CD8 T cell response. Whereas the response to CSP increased with repeated sporozoite exposures in BALB/c mice, the response to L3 was not strongly recalled by subsequent sporozoite exposures. The L3-specific T cells were functionally cytotoxic and could be re-expanded by a non-sporozoite booster in the form of *Listeria monocytogenes* recombinantly expressing the L3 epitope [34]. Although single dose immunizations with attenuated sporozoites do not usually lead to sterile protection in mice [35], a single immunization does achieve a significant level of partial protection, as measured by liver burden assessments [34]. Subsequent immunizations further increase this protective effect, indicative of gradual acquisition of sterile protective immunity. At the vaccination stage, gradually acquired protection leading to significant reductions in liver burden also correlated with significant reductions in the L3 antigen load [34]. Since L3 was mostly expressed in the liver and later erythrocyte stages but not the sporozoite stage, the acquisition of immunity against the sporozoites used for vaccination were reducing the immunogenicity of the vaccine for antigens that needed to be expressed de novo in the hepatocyte [34]. Here, a series of known *Plasmodium berghei* antigens were examined in C57BL/6 mice multiply immunized with sporozoites to determine if the distinction between boostable versus non-boostable responses was generalizable beyond BALB/c mice. In addition, heterologous cross-species/strain immunization was tested to determine if this modified the immunogenicity of shared sporozoite antigens.

## Methods

### Mice

All animal studies were approved by the University of Washington Institutional Animal Care and Use Committee (IACUC protocol 4317-01). Female BALB/cj and C57BL/6 mice were obtained from Jackson Laboratories (Bar Harbor, ME, USA). A breeding pair of C57BL/6-derived μMT mice (B6.129S2-Ighmtm1Cgn/J) were also obtained from Jackson Laboratories and were bred at the University of Washington. All mice were housed in standard IACUC-approved small animal facilities and used in compliance with IACUC-approved protocols.

### Sporozoite vaccination and challenge

WT *Plasmodium yoelii* 17XNL, WT *P. berghei* ANKA and *P. yoeliifabb/f*^−/−^ (GAP) sporozoites were obtained by salivary gland dissection from *Anopheles stephensi* mosquitoes reared at the Research Insectary at the Center for Infectious Disease Research (formerly Seattle Biomed, Seattle, WA, USA). Where indicated, RAS were generated by exposure to 10,000 rads using an X-ray irradiator (Rad-Source, Suwanee, GA, USA). Unless stated otherwise, RAS, GAP and WT sporozoites were administered by intravenous tail vein injection in a volume of 150 μL. Multiply immunized mice were vaccinated at three-week intervals. Where indicated, sporozoites were purified using the Accudenz gradient method [36]. For challenge, mice were intravenously challenged with 1000 or 10,000 WT sporozoites.

### Ex vivo IFNγ enzyme-linked immunosorbent spot (ELISPOT) assays

For ELISPOTs, peptides corresponding to known CD8^+^ T cell epitopes (1 µg/mL final) were combined with 1 × 10^6^ murine splenocytes by murine interferon-γ (IFNγ) ELISPOT (Affymetrix, Santa Clara, CA, USA) and cultured in antibody-coated ELISPOT plates 18 h at 37 °C as previously reported [34]. *Plasmodium berghei* peptides used to assess responses included those for PbTRAP_130–138_ (H2-D^b^-restricted SALLNVDNL from PBANKA_1349800 TRAP [37]), PbS20_318–326_ (H2-K^b^-restricted VNYSFLYLF from PBANKA_1429200 sporozoite-specific gene 20 (S20) [37]), PbGAP50_41–48_ (SQLLNAKYL from PBANKA_0819000 glideosome-associated protein 50 (GAP50) [38, 39]), PbF4 (EIYIFTNI from PBANKA_0416600 replication protein A1 [40]), PbNCY (NCYDFNNI from PBANKA_0714500 [41]) and PbCSP_245–253_ (H2-K^d^-restricted SYIPSAEKI from PBANKA_0403200 circumsporozoite protein (CSP). *Plasmodium yoelii* peptides used to assess responses included those for PyCSP_280–288_ (H2-K^d^-restricted SYVPSAEQI from PY03168) and PyL3 (H2-K^d^-restricted GYKSGMSHI from PY05881 [34]). All gene identifiers refer to PlasmoDB names [42]. ELISPOT plates were developed using a colorimetric substrate as reported [34]. All ELISPOT wells were tested in two to three wells per mouse per antigen and cumulative ELISPOT data were evaluated using the mean spot forming units (SFU) per million splenocytes for each animal.

### Bioinformatics analysis of class I peptide-sized homology

FASTA files of all protein sequences were downloaded from PlasmoDB [42] for *Plasmodium* species (*P. falciparum* 3D7, *P. vivax* Sal-1, *P. berghei* ANKA, *P. yoelii* 17XNL). Comparisons were made between proteins of *P. falciparum* 3D7 and *P. vivax* Sal1 and between the proteins of *P. berghei* ANKA and *P. yoelii* 17XNL. For each pair of species, an exhaustive search was carried out on the full protein sets of the two species to find all common 8-amino acid sequences. The search strategy was to loop through the first protein set using 8-amino acid by 8-amino acid comparisons. For the second protein set, proteins were concatenated end-to-end 100 at a time, with a separator symbol ($) between successive proteins. The concatenated string was searched using a sorted suffix array (reviewed in [43]). Matching 8-amino acid regions were then extended to find the longest exact matching peptide of length ≥8 amino acids. This strategy was implemented in a Python program. Data consisting of the common peptide sequence, species 1 protein identifier and species 2 protein identifier were filtered to include syntenic orthologues only and further categorized by available expression data (e.g., sporozoite or liver stage). In addition, highly repetitive peptides such as pure runs or nearly pure runs of a single amino acid were removed from the database—this last criterion was especially useful for *P. falciparum* where asparagine repeats are extremely common [44]. Expression data consisted of mass spectrometry data from several large published datasets [10, 45–47] transformed by syntenic orthology to generate lists of proteins where the protein of interest or its syntenic orthologue was identified as a sporozoite (spz) and/or liver stage protein. Proteins were included if mass spectrometry data demonstrated at least one peptide and one spectra minimum. Stage-specific datasets included *P. berghei* and *P. yoelii* salivary gland sporozoites [45], *P. falciparum* salivary gland sporozoites [10, 45, 46] and *P. yoelii* liver stage proteins [47]. The lengths of the homologous peptides were recorded and percent homologous sequence compared to the total encoded protein sequence for each species.

### B cell depletion experiments

Anti-CD20 antibody (clone 5D2 IgG2a) was provided by Genentech. BALB/cj mice were injected with 250 μg of anti-CD20 one day prior to primary immunization with 1 × 10^4^ purified *P. yoelii* RAS. Four weeks later, mice were injected again with anti-CD20 and then administered a second homologous dose of 1 × 10^4^ purified *P. yoelii* RAS 2 days later. B cell depletion was confirmed by evaluating peripheral blood for the presence of B220^+^ cells in the single cell lymphocyte gate on a Canto flow cytometer (BD, Franklin Lakes, NJ, USA).

### Liver stage *Plasmodium* 18S rRNA assay

At the indicated time post-challenge, mice were sacrificed, and half of the total liver was excised and pulverized by bead beating in 5 mL NucliSENS lysis buffer (bioMérieux, Durham, NC, USA). Total RNA was extracted by processing 100 μL of the NucliSENS buffer-treated sample diluted 1:10 in NucliSENS lysis buffer on the EasyMag system (bioMérieux) as described for whole blood [48]. In some experiments, livers were pulverized in 5 mL Trizol (Life Technologies/Invitrogen, Carlsbad, CA, USA) and total RNA was Trizol extracted as described [34]. RNA was subjected to RT-PCR using the One Step AgPath RT-PCR kit (Invitrogen) using a predesigned HEX-labelled mouse GAPDH RT-PCR assay (IDT Inc, Coralville, IA, USA) multiplexed with a Pan-*Plasmodium* 18S rRNA assay. The Pan-*Plasmodium* 18S rRNA reagents consisted of a CalFluor Orange560-labelled Pan-*Plasmodium* probe (5′[CAL Fluor Orange 560]-ACCGTCGTAATCTTAACCATAAACTA[T(BHQ1)]GCCGACTAG-3′; Biosearch Technologies, Navato, CA, USA) and adjacent primers (forward 5′-AAAGTTAAGGGAGTGAAGA-3′; reverse 5′-AAGACTTTGATTTCTCATAAGG-3′) under the following conditions (45 °C for 20 min, 95 °C for 15 min and 45 cycles of 95 °C for 20 s, 50 °C for 30 s, 60 °C for 30 s) on a CFX96/1000C real-time PCR machine (Biorad, Hercules, CA, USA). Data were normalized to mouse GAPDH and transformed to relative log_10_ values to compare log_10_ copy number reduction in *Plasmodium* 18S rRNA versus the control group.

## Results

### Most malaria-specific CD8^+^ T cell responses contract with repeated whole sporozoite immunization except for those targeting protective TRAP and CSP antigens

**Fig. 1:**
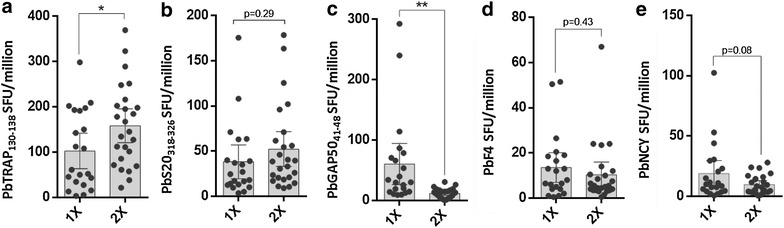
Multiple *Plasmodium* *berghei* RAS immunizations induce T cell responses with characteristically expanding or contracting frequencies in the C57BL/6 models of *Plasmodium* sporozoite immunization. C57BL/6 mice immunized once (1X) or twice (2X) with 1-2x10^4^ *P. berghei* ANKA RAS respond to **a** PBANKA_1349800 TRAP (H2-Db-restricted TRAP130-138 SALLNVDNL), **b** sporozoite-specific gene 20 (S20) PBANKA_1429200 (H2-Kb restricted S20318-326 VNYSFLYLF), **c** PBANKA_0819000 secreted acid phosphatase glideosome-associated protein 50 (GAP50) (H2-Db restricted GAP5041-48 SQLLNAKYL), **d** PBANKA_0416600 replication protein A1 (PbF4 peptide EIYIFTNI) and **e** PBANKA_0714500 (H2-Kb-restricted PbNCY peptide NCYDFNNI). *Bars* display mean value and *error bars* show the 95 % confidence interval; *p < 0.05, **p < 0.01, ***p < 0.001, ****p < 0.0001, Students t-test. For all peptides, splenocytes from naïve mice showed < 2 SFU/million

To determine if the previous finding [34] of expansion of CSP-specific T cells and contraction of L3-specific T cells in BALB/c mice was generalizable in other murine *Plasmodium* models, known *P. berghei* H2^b^ epitopes were tested to determine the frequency of CD8^+^ T cell responses in sporozoite-immunized C57BL/6 mice. C57BL/6 mice were immunized one to three times with 1–2 × 10^4^*P. berghei* ANKA RAS at three-week intervals. IFNγ responses to PbTRAP_130–138_, PbS20_318–326_, PbGAP50_41–48_, PBANKA_0416600 (PbF4), and PBANKA_071450 (PbNCY) were assessed by splenocyte ELISPOT 6 days after the final immunization. Responses to PbTRAP_130–138_ (Fig. 1a) and to some extent PbS20_318–326_ (Fig. 1b) trended toward increased frequency with multiple immunizations whereas responses to PbGAP50_41–48_ (Fig. 1c), PbF4 (Fig. 1d) and PbNCY (Fig. 1e) contracted. These findings are similar to what is found in BALB/cj mice multiply immunized with *P. yoelii* RAS (1–2 × 10^4^ spz/dose) where the CSP T cell population expands compared to that of the L3-specific T cells (Additional file 1: Figure S1 and [34]). These collective results show that in two mouse strains and with two parasite species, T cell responses to preformed antigens like CSP and TRAP can stabilize and even expand in numbers in the setting of repeated sporozoite exposure whereas most other antigen-specific T cells contracted. Most proteins whose responding T cell populations contract are either absent or mostly absent from sporozoites and/or are more highly expressed in newly forming liver stage parasites.

### Heterologous cross-species immunization has the potential to generate a more diverse T cell repertoire

*Plasmodium* species share considerable amino acid homology. Despite this, most immunization-challenge models have consisted almost exclusively of homologous immunization followed by homologous challenge. Recently, some groups have begun to evaluate homologous *P. falciparum* (single strain) immunizations followed by heterologous challenge with a different *P. falciparum* strain in CHMI studies [49].

Since heterologous challenge circumvents some antigen-specific immune responses [50], it was possible that a heterologous (cross-species) immunization regimen could expand CD8^+^ T cell responses against shared epitopes more so than a homologous regimen. To gauge the potential breadth of shared Class I epitopes, bio-informatic analyses were performed to look for interspecies homology of syntenic orthologues using a minimum homology length of 8 aa, the shortest length of a typical Class I MHC-binding peptide. First, coding sequences for all predicted proteins [*P. yoelii* 17XNL 7724 (3.3 × 10^6^ total aa), *P. berghei* ANKA 4952 (3.4 × 10^6^ total aa), *P. falciparum* 5398 (4.1 × 10^6^ total aa), *P. vivax* Sal-1 5530 (3.9 × 10^6^ total aa)] were filtered to include only syntenic orthologous pairs. For *P. yoelii* 17XNL/*P. berghei* ANKA, there were 4191 pairs comprising 4166 *P. yoelii* 17XNL proteins of 2.4 × 10^6^ total aa and 4075 *P. berghei* ANKA proteins of 2.9 × 10^6^ total aa. For *P. falciparum*/*P. vivax* Sal1, there were 4777 pairs comprised of 4638 unique *P. falciparum* proteins of 3.6 × 10^6^ total aa and 4584 *P. vivax* Sal1 proteins of 3.4 × 10^6^ total aa) (Additional file 2).

The predicted amino acid sequences for syntenic orthologous pairs were searched for homology of ≥8 contiguous aa, corresponding to the minimum length of typical Class I MHC peptides (Table 1). *Plasmodium yoelii* 17XNL/*P. berghei* ANKA demonstrated a higher degree of homology than *P. falciparum*/*P. vivax* Sal1, with 67,684 homologous peptides in the former and just 41,417 in the latter across all life cycle stages. For sporozoites proteins, 41,424 peptides were conserved for *P. yoelii*/*P. berghei* compared to 28,793 *P. falciparum*/*P. vivax* and at the liver stage 7659 were conserved for *P. yoelii*/*P. berghei* and 7813 for *P. falciparum*/*P. vivax* (Table 1). The higher degree of conservation was also reflected in longer mean lengths of homologous peptides (p < 0.0001, Student’s two-sided t-test), longer maximum length peptides (*P. yoelii*/*P. berghei* 926 aa versus *P. falciparum*/*P. vivax* 438 aa) and a larger percentage of total syntenic orthologous amino acids conserved in MHC-binding peptide-length windows for *P. berghei*/*P. yoelii* (Additional file 3) compared to *P. falciparum*/*P. vivax* (Additional file 4). The relative per cent conservation of syntenic sequence increased in sporozoite proteins and increased further in LS proteins, suggesting that these proteins are less variable between species. As expected, in this analysis the H2-K^d^-binding CSP epitopes are not conserved between *P. berghei* and *P. yoelii* (two amino acid differences) and were not included, whereas the L3 epitope is completely conserved between *P. berghei* and *P. yoelii* species. As expected, the conserved peptides tested here (L3, S20, GAP50, F4, NCY) were present in the shared dataset, whereas the CSP and TRAP epitopes were not. Along these lines, both *P. yoelii* and *P. berghei* sporozoites (spz) could prime CD8^+^ T cell responses to the shared L3 epitope in Balb/cj mice, whereas only *P. yoelii* spz could prime responses to the PyCSP epitope. Similarly, only *P. berghei* spz could prime responses to the PbCSP epitope (Additional file 5: Figure S2). This finding is in agreement with previous work showing that CTLs that target the PbCSP epitope can protect *P. berghei* spz challenge but not against *P. yoelii* spz [12].

**Table 1:** Shared 8-mer linear peptidome of rodent and human *Plasmodium* parasites

Pairing	Stage	Homologous windows (#≥8 aa)	Mean length (aa)	Max length (aa)	Total aa conserved in ≥8 aa windows	% of syntenic proteome^a^ (%)
Py/Pb	All	67,684	25.5	926	1.72 × 10^6^	70.5 % Py/59.3 % Pb
Pf/Pv	All	41,417	15.7	438	6.49 × 10^5^	18.1 % Pf/18.9 % Pv
Py/Pb	Spz	41,424	26.9	926	1.12 × 10^6^	All: 46.5 % Py/38.4 % Pb Spz: 76.2 % Py/61.6 % Pb
Pf/Pv	Spz	28,793	16.4	438	4.73 × 10^5^	All: 13.2 % Pf/13.8 % Pv Spz: 20.8 % Pf/13.8 % Pv
Py/Pb	LS	7659	34.8	831	2.67 × 10^5^	All: 10.9 % Py/9.2 % Pb LS: 79.5 % Py/77.9 % Pb
Pf/Pv	LS	7813	19.3	438	1.51 × 10^5^	All: 4.2 % Pf/4.4 % Pv LS: 38.6 % Pf/39.3 % Pv

Pb, *P. berghei* ANKA; Py, *P. yoelii* 17XNL; Pf, *P. falciparum* 3D7; Pv, *P. vivax* Sal1; aa, amino acids

^a^Comparison to all stage or stage-specific syntenic proteomes. Total aa for all stages (Py 2.44 × 10^6^ aa; Pb 2.90 × 10^6^ aa; Pf 3.58 × 10^6^ aa; Pv 3.42 × 10^6^ aa), sporozoite stage (Py 1.46 × 10^6^ aa; Pb 1.81 × 10^6^ aa; Pf 2.27 × 10^6^ aa; Pv 2.19 × 10^6^ aa) and liver stage (Py 3.36 × 10^5^ aa; Pb 3.42 × 10^5^ aa; Pf 3.83 × 10^5^ aa; Pv 3.90 × 10^5^ aa)

### Heterologous cross-species immunizations achieve larger secondary liver infections than homologous immunizations

**Fig. 2:**
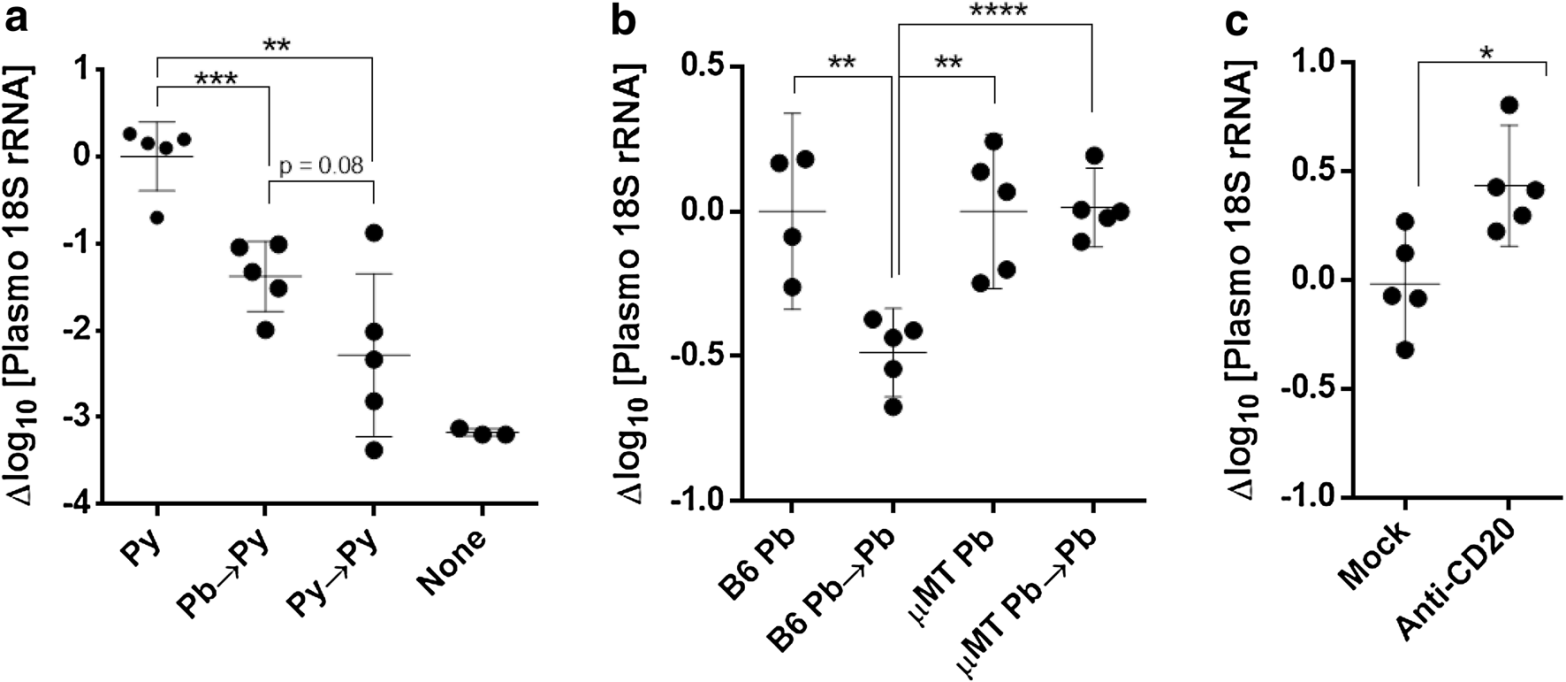
Heterologous cross-species immunization increases secondary liver burden compared to homologous immunization in part by circumventing homologously directed antibody responses. **a** Liver burden at 44 h after single (Py), homologous (Py → Py) or heterologous (Pb → Py) RAS immunizations of BALB/cj mice compared to uninfected animals (none). **b** Liver burden at 24 h after final immunization of WT C57BL/6 or μMT mice with either single (Pb) or double (Pb → Pb) homologous RAS regimens. **c** Liver burden at 24 h after homologous RAS immunization of BALB/cj mice mock-treated or treated with anti-CD20 antibodies to deplete B cells. *p < 0.05,**p < 0.01, ***p < 0.001,****p < 0.0001, Students t-test. All doses were 1 × 10^4^ RAS. *Plasmodium* 18S rRNA content was normalized to the host GAPDH mRNA and differences are expressed in log_10_ changes in parasite 18S rRNA concentration relative to the single exposure control average

In recent work, repeated homologous immunization was shown to progressively reduce liver stage burdens—even a single *P. yoelii* RAS immunization reduced the next immunization liver burden by >90 % [34]. To determine if liver infection was greater following heterologous cross-species immunization, BALB/cj mice were immunized homologously or heterologously and liver burden was measured by *Plasmodium* 18S rRNA RT-PCR at 44 h following the second immunization. Both the heterologous (*P. berghei* → *P. yoelii*) and homologous (*P. yoelii* → *P. berghei*) second doses resulted in smaller magnitude liver infections compared to a single dose of sporozoites given to naïve mice (*P. yoelii*) (Fig. 2a). Homologously immunized mice generally had extremely low liver burdens at levels that approached uninfected mice in some cases. Heterologously immunized mice showed a smaller reduction in liver burden compared to homologously immunized mice (Fig. 2a). These results indicated that there could be more antigen in the livers of heterologously immunized mice that could potentially stimulate greater secondary T cell responses against shared epitopes, in particular against antigens not normally boosted by homologous immunizations.

Previous work on cross-species protection in homologously immunized C57BL/6 mice suggested that such protection was T cell dependent since adoptively transferred antisera generated by homologous immunization could not confer protection against cross-species challenge [50]. To determine if heterologous immunization resulted in larger liver burdens by circumventing the antibody responses stimulated by the priming immunization, several experiments were conducted on the C57BL/6 and BALB/c backgrounds. The following B-cell deficient mouse experiments were measured by liver-stage RT-PCR at 24 h post-immunization in order to separate effects of cell-mediated killing and antibody-mediated blockade of invasion. First, WT C57BL/6 mice and C57BL/6-derived μMT mice were compared in homologous *P. berghei* RAS immunization experiments. μMT mice lack mature B cells and do not make antibody responses. WT C57BL/6 and μMT mice were immunized with *P. berghei* RAS once or twice and the parasite liver burden was measured at 24 h after the last immunization as a measure of initial liver-stage infection. At 24 h post-challenge, homologously immunized B6 mice showed a reduction in liver burden compared to singly exposed animals (Fig. 2b). In contrast, there was no such reduction in B-cell deficient μMT mice regardless of one or two exposures (Fig. 2b). This finding suggests that a single exposure to sporozoites elicits antibody-mediated ‘debulking’ of subsequent immunizations in immunocompetent C57BL/6 mice. μMT mice could not be used to assess whether the resulting increase in liver burden increased overall immunogenicity because repeated immunizations led to the development of small brittle spleens that demonstrated unacceptably high lymphocyte mortality (>80 %) upon splenocyte harvest. To test the role of antibodies in BALB/c mice, anti-CD20 antibodies (kind gift of Genentech) were used to eliminate B cells before each of two *P. yoelii* RAS immunizations in BALB/cj mice. Anti-CD20 antibody-treated mice showed no demonstrable B cells by anti-B220 staining compared to control mice (Additional file 6: Figure S3). Antibody-treated, B cell-deficient BALB/c mice showed a more than twofold increase in parasite liver burden (+0.45 log_10_ copies *Plasmodium* 18S rRNA) at 24 h post-challenge compared to untreated mice (Fig. 2c). Changes in liver burden observed in B-cell deficient mice (through genetic- and antibody-mediated approaches) show that antibodies reduce the liver burden in homologously immunized mice. These findings support the idea that heterologous immunization circumvents homologous antibody-dependent protection that otherwise would debulk the intended liver infection.

### Heterologous immunization recalls some but not all CD8^+^ T cell responses that normally contract following homologous immunizations

**Fig. 3:**
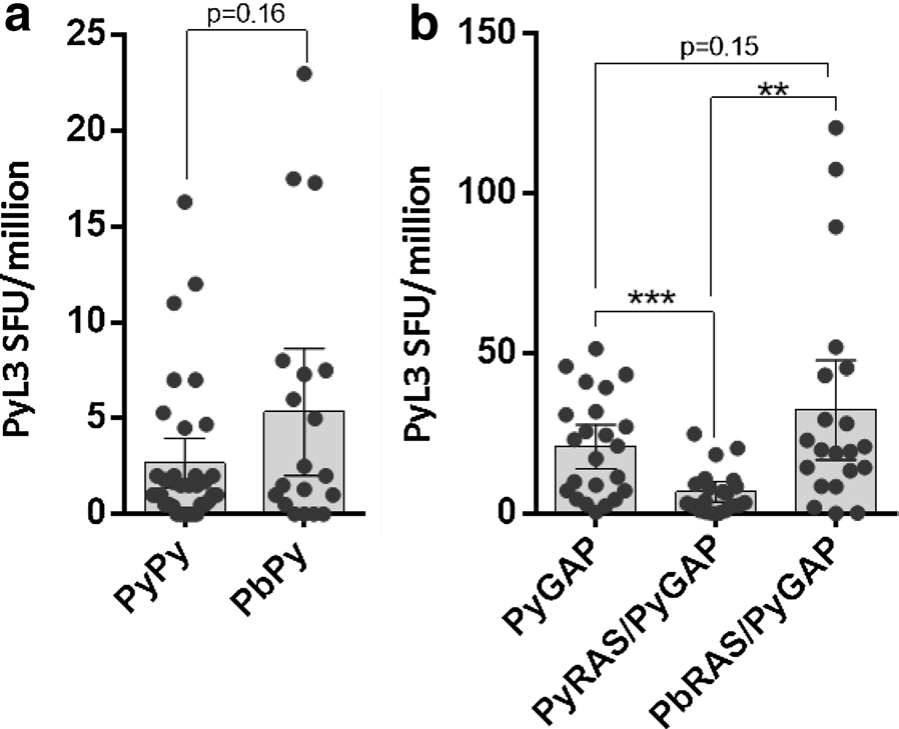
Heterologous immunization with late-arresting attenuated sporozoites leads to higher secondary CD8^+^ T cell responses to the L3 antigen in BALB/cj mice. **a** L3-specific responses in BALB/cj mice immunized homologously with *P. yoelii* RAS (Py/Py) or heterologously with *P. berghei* RAS followed by *P. yoelii* RAS (Pb/Py). Heterologous immunization showed a small non-significant increase in L3-specific responses. **b** L3-specific responses in BALB/cj mice immunized with *P. yoelii fabb/f* ^−/−^ GAP (PyGAP) or homologously with *P. yoelii* RAS followed by *P. yoelii* GAP (PyRAS/PyGAP) or heterologously with *P. berghei* RAS followed by *P. yoelii* GAP (PbRAS/PyGAP). Homologous immunization showed the typical contraction of the L3-specific response, whereas heterologous exposure resulted in a significant increase relative to homologous levels. In some cases, mice mounted extremely strong L3 responses following secondary heterologous exposure that were never seen following primary exposure. **p < 0.01, ***p < 0.001, Student’s t-test. All injections were 1 × 10^4^ sporozoites by intravenous route

With the hypothesis that heterologous cross-species immunization could boost the CD8^+^ T cell repertoire against shared antigens more than conventional homologous regimens, mice were immunized with two-dose sporozoite regimens consisting of *P. yoelii* 17XNL RAS and/or *P. berghei* ANKA RAS and measured CD8^+^ T cell responses to known *P. yoelii* and *P. berghei* antigens 6 days later. Heterologously immunized BALB/cj mice (*P. berghei* RAS → *P. yoelii* RAS) recalled responses to L3 at marginally higher frequencies than by homologous vaccination (*P. yoelii* RAS → *P. yoelii* RAS), although the effect was not statistically significant and the overall magnitude of these responses was extremely low (Fig. 3a). PyCSP-specific T cell responses were not increased by heterologous immunization, consistent with the fact that PyCSP and PbCSP H2-K^d^ epitopes differ at two amino acids as noted above. Since late-arresting sporozoites such as *P. yoeliifabb/f*^−*/*−^ GAP are more ‘fit’ and develop longer than *P. yoelii* RAS, it was possible that prolonged pre-erythrocytic development of the *P. yoelii* GAP would result in more antigen expression. *P. yoeliifabb/f*^−*/*−^ GAP is a late-arresting GAP attenuated by deletion of FabB/F, an important enzyme in *Plasmodium* fatty acid synthesis [51]. BALB/cj mice were immunized with *P. yoelii* GAP alone or with homologous (*P. yoelii* RAS → *P. yoelii* GAP) or heterologous (*P. berghei* RAS → *P. yoelii* GAP) regimens. While L3 responses contracted in homologously immunized mice compared to singly exposed mice, heterologous immunization recalled responses at significantly higher frequencies than the homologous regimen (Fig. 3b). The overall magnitude of the L3 response at primary GAP and heterologous RAS → GAP secondary endpoints was also substantially higher than in RAS-only immunizations. This suggests that heterologous immunization can achieve higher secondary liver burdens and that use of a late-arresting sporozoite at the secondary booster vaccination can likely lead to higher L3 antigen concentrations that induce higher secondary L3-specific T cell frequencies.

**Fig. 4:**
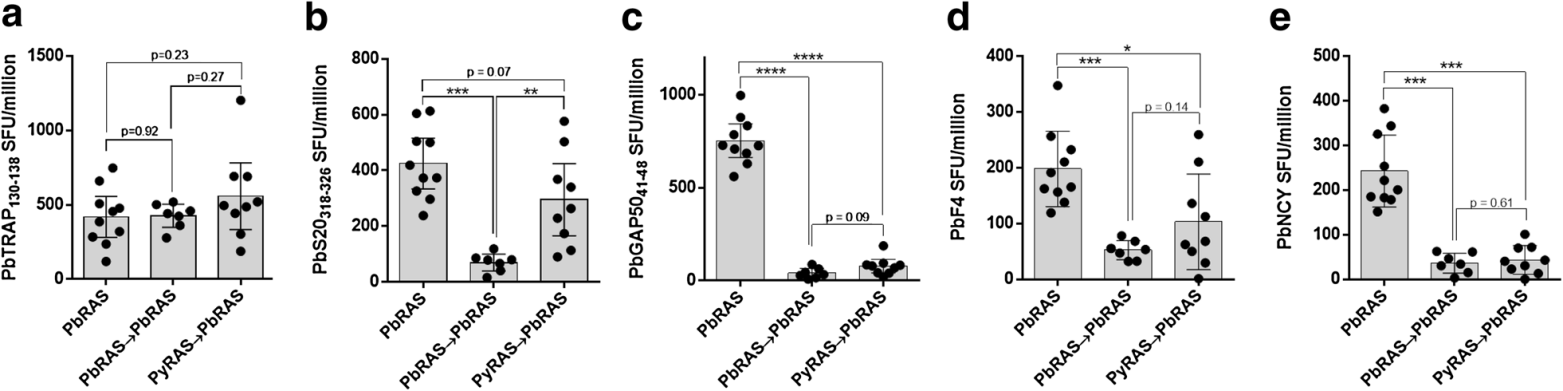
Heterologous immunization with a high-dose RAS booster in C57BL/6 mice can be used to distinguish between recalled and non-recalled responses. Antigen-specific responses in C57BL/6 mice immunized once with 8 × 10^4^ *P. berghei* RAS (PbRAS), homologously with 1 × 10^4^ *P. berghei* RAS followed by 8 × 10^4^ *P. berghei* RAS (PbRAS → PbRAS) or heterologously with 1 × 10^4^ *P. yoelii* RAS followed by 8 × 10^4^ *P. berghei* RAS (PyRAS → PbRAS). Antigens included PbTRAP (**a**), PbS20 (**b**), PbGAP50 (**c**), PbF4 (**c**) and PbNCY (**e**); additional identifying details are in Fig. 1 and “Methods” section. *Bars* display mean value and *error bars* show the 95 % confidence interval; *p < 0.05, **p < 0.01, ***p < 0.001, ****p < 0.0001, Student’s t-test

In C57BL/6 mice, most well-studied antigens are *P. berghei* derived, which meant that the secondary immunization in heterologous C57BL/6 experiments needed be *P. berghei* to ensure fair comparison to singly exposed mice. As there are no late-attenuated *P. berghei* parasite lines widely available, heterologous experiments in C57BL/6 mice aimed to achieve higher secondary antigen concentrations by instead increasing the dose of RAS administered at the second exposure. WT C57BL/6 mice were immunized with nothing or with 1 × 10^4^ purified *P. yoelii* RAS or 1 × 10^4^ purified *P. berghei* RAS. Three weeks later, all mice were immunized with 8 × 10^4^ purified *P. berghei* RAS and CD8^+^ T cell responses to known antigens were assessed 6 days later. PbTRAP_130–138_-specific responses did not decline after a second homologous or heterologous immunization (Fig. 4a), consistent with the previously observed maintenance or even boosting of this cell population with re-exposure at lower booster doses. In this high-dose booster model and in contrast to what was seen in the low dose *P. berghei* RAS booster (Fig. 1b), responses to PbS20_318–326_ contracted on secondary homologous exposure, but could be recalled by a high dose heterologous booster (Fig. 4b). Responses to PbGAP50_41–48_ (Fig. 4c), PbF4 (Fig. 4d) and PbNCY (Fig. 4e) were all highly induced by a large single *P. berghei* RAS exposure but could not be recalled by either homologous or heterologous double exposures, findings consistent with the low dose homologous exposure (Fig. 1c–e). These data begin to categorize the CD8^+^ T cell repertoire induced by *Plasmodium* sporozoites into responses primed but not recalled by re-exposure, primed and recalled by homologous re-exposure or primed and recalled by heterologous but not homologous re-exposure.

## Discussion

Although repeated homologous sporozoite immunization protects humans from WT *P.falciparum* challenge, the regimen that achieves protection requires four to five intravenous doses of 1.35 × 10^5^ irradiated, cryopreserved sporozoites at each dose [1]. Less attenuated sporozoites such as late-arresting GAPs [4] or WT sporozoites administered under anti-malarial drug coverage [2] may have a dose-sparing effect but GAP vaccines have not yet been manufactured in a vialed format. Vialed, cryopreserved WT sporozoites under a concurrent drug treatment-vaccination approach known as CVac [52] are being developed but may require optimization. At this time, it is difficult to make a head-to-head comparison about the immunogenicity of PfSPZ, GAP and/or CVac-type approaches because optimal vaccination schedules have not been finalized for any of these approaches. Modifications that reduce dosing frequency and/or dosage while increasing immunogenicity (and ultimately efficacy) may improve delivery of whole sporozoite vaccine product. This study was undertaken in the mouse model to evaluate sporozoites vaccination modifications that could generally enhance the immunogenicity of sporozoite-based vaccines.

In previous work in the BALB/cj model [34], two model *P. yoelii*-derived antigens were found to induce very different CD8^+^T cell responses upon repeated sporozoite immunizations. The well-studied CSP antigen-induced cytotoxic CD8^+^ T cells that increased in frequency with multiple doses of 1 × 10^4^ sporozoites (RAS or GAP). In contrast, the L3 ribosomal protein induced a comparable number of cytotoxic CD8^+^ T cells as CSP upon primary immunization but these responses did not increase and generally contracted with multiple immunizations. Here, the same pattern of secondary recall or contraction was observed for T cell responses to a panel of known *P. berghei* epitopes in C57BL/6 mice. PbTRAP_130-138_ (SALLNVDNL) is an H2-D^b^-restricted cytotoxic immunodominant CD8^+^ T cell epitope in C57BL/6 mice derived from PBANKA_1349800 TRAP that contributes to protection against liver-stage infection [37]. PbS20_318–326_ (VNYSFLYLF) is an H2-K^b^-restricted but non-cytotoxic, non-protective CD8^+^ T cell epitope derived from PBANKA_1429200 sporozoite-specific gene 20 (S20) [37]. PbGAP50_41–48_ (SQLLNAKYL) is derived from PBANKA_0819000 glideosome-associated protein 50 known as GAP50 and targeted by cytotoxic CD8^+^ T cells [38, 39] but is not protective [53]. PbGAP50_41–48_-specific cells have amongst the highest precursor frequency identified for any T cell with ~2200 PbGAP50_41–48_-specific cells per animal [54]. PbF4 (EIYIFTNI) is derived from PBANKA_0416600 replication protein A1 and is a blood stage CD8^+^ epitope [40]. Recently, T cells responding to Pb1, F4 and another epitope from PBANKA_113700/bergheilysin (IITDFENL) were shown to damage the blood–brain barrier in a murine model of *P. berghei*-induced cerebral malaria [39]. PbNCY (NCYDFNNI) is derived from PBANKA_0714500 and is the target of so-called ‘PbT1′ T cells that have been shown to respond to *P. berghei* sporozoites and infected erythrocytes [41]. This antigen is encoded on sporozoites and PbT1 cells are able to protect mice from liver-stage challenge infection following a spleen-centered CD8α^+^ dendritic cell-dependent immune response, indicating that the antigen may be partly cross-presented [41].

Across both BALB/c and C57BL/6 backgrounds, the collective data now show that antigen-specific CD8^+^ T cell responses to PyL3, PbS20_318–326_, PbGAP50_41–48_, PbF4, and PbNCY contract despite repeated immunization whereas responses to protective TRAP and CSP antigens remain stable or even expand. Responses that contract do not exclusively target late liver stage antigens. As previously hypothesized [34], the failed secondary expansion of cells targeting proteins like L3, F4 and NCY may be explained by the ‘debulking’ of the secondary immunization dose achieved by the poly-specific immune response. However, PbS20 and PbGAP50 are both present in higher quantities in the sporozoite itself (Table 2), albeit at much lower concentrations than CSP and TRAP. The T cells responding to these antigens are unlikely to have functional defects that preclude their secondary expansion since L3 [34], PbGAP50 [53] and NCY [41] have all been shown to expand to extremely high T cell frequencies with non-parasite booster vaccines.

**Table 2:** Comparison of immunogenicity data to available mass spectrometry-based expression data

Antigen	Epitope	MHC	Protective?	Conserved in Pb/Py?	Recall?		No. spectra in Py/Pf spz^b^ (Py rank/Pf rank)	No. spectra from Py/Pf spz surface?^b^ (Py rank/Pf rank)	Δ Transcription (24/40 h LS *vs* sgSPZ)^c^
					Hom	Het			
PyCSP	SYVPSAEQI	H2K^d^	Yes	No^a^	Yes	NA	549/1460 (22/5)	166/140 (1/1)	ND/ND
PbTRAP	SALLNVDNL	H2D^b^	Yes	No^a^	Yes	NA	1437/2164 (13/4)	16/1 (10/22)	−1.91/−3.01
PbS20	VNYSFLYLF	H2 K^b^	No	Yes	No	Yes	536/652 (61/44)	ND/5 (NA/11)	ND/ND
PyL3	GYKSGMSHI	H2K^d^	No	Yes	No	Yes	291/82 (89/402)	ND/ND (NA/NA)	3.00/2.88
PbGAP50	SQLLNAKYL	H2D^b^	No	Yes	No	No	390/584 (51/24)	ND/ND (NA/NA)	1.31/–1.08
PbF4	EIYIFTNI	H2^b^	No	Yes	No	No	46/84 (630/481)	ND/ND (NA/NA)	ND/ND
PbNCY	NCYDFNNI	H2^b^	No	No^d^	No	No	ND/ND (NA/NA)	ND/ND (NA/NA)	ND/ND

*Hom* homologous; *Het* heterologous; *NA* not applicable; *ND* not detected

^a^PyCSP SYVPSAEQI vs. PbCSP SYIPSAEKI; PyTRAP SALLVVDTL vs. PbTRAP SALLNVDNL, amino acid differences underlined

^b^Number of spectra detected for Py (*P. yoelii*) and Pf (*P. falciparum*) orthologues of the tested antigen based on mass spectrometry of sporozoites as described in [45]

^c^Change in mRNA transcription for 24 h LS:sgSPZ or 40 h LS:sgSPZ based on [47] and PlasmoDB [42]

^d^Py protein truncated compared to Pb protein

To address whether a simple ‘debulking’ model explained the contraction of responses to S20, GAP50, F4, and NCY, heterologous immunization was tested to determine if this approach could secondarily expand these specific T cell populations. Bio-informatic analysis demonstrated that there is a considerable amount of protein sequence homology that constitutes peptides of sufficient length to be Class I MHC targets. To pursue this approach experimentally, BALB/cj mice immunized with *P. berghei* RAS then *P. yoelii* RAS achieved larger liver burdens upon secondary immunization as compared to mice immunized twice with *P. yoelii* RAS. Although a major goal of vaccine developers is to produce a malaria vaccine that achieves cross-species protection, the imperfect cross-species immunity achieved after a single immunization with just one species means that the vaccine ‘take’ upon secondary heterologous immunization is improved. This debulking is at least partially due to antibodies directed against homologous parasites since circumventing such antibodies can increase liver burden upon secondary vaccination. The T cell repertoire is potentially extremely large and could benefit from secondary boosting of responses to shared epitopes. Although L3 and PbS20 are both non-protective, these antigens could be recalled by heterologous but not homologous immunization. The assumption is that these antigens are emblematic of a class of antigens that do not re-expand due to debulking of the secondary immunization by the immunity achieved by primary immunization. If this class of antigens contains as yet undiscovered protective targets, heterologous immunization could accelerate their discovery. L3 responses were more vigorously recalled by secondary heterologous immunization with a late-arresting GAP compared to the early-arresting RAS approach. Thus, a more ‘fit’ parasite may also improve recall of liver-stage antigens. It seems unlikely that the degree of attenuation would profoundly affect responses to antigens that are primarily pre-formed in the sporozoite. This may explain why abundant pre-formed CSP and TRAP antigens expand responses while less abundant pre-formed antigens like PbS20 and PbGAP50 do not. The strong primary responses to PbS20 and PbGAP50 and lack of secondary homologous responses also suggest that these responses probably depend on sufficient hepatocyte infection. Heat-killed sporozoites can induce responses to CSP but at considerably lower frequencies than with live sporozoites [34, 55], which also implies that responses to CSP also depend on liver infection. Responses to the CSP and TRAP epitopes tested here did not benefit from heterologous immunization because there are amino acid differences between the species that preclude cross-reactive responses by species-specific T cells. Heterologous RAS immunization increased the secondary response to the conserved PbS20 antigen. For some conserved antigens however (PbGAP50, F4, NCY), even heterologous RAS immunization did not lead to secondary recall, suggesting that these antigens may not be of sufficient quantity even when the liver burden is modestly increased by heterologous exposure.

How much of the Pb20 and GAP50 antigens come from sporozoite versus intrahepatic liver expression is unknown. Mass spectrometry data shows that peptides from *P. falciparum* S20 can also be found on the surface of sporozoites [45] and it is possible that secondary recall is enhanced by antigen surface exposure in addition to heterologous RAS exposure. Little is known about the role of GAP50 in the sporozoite. Although GAP50 peptides are found in sporozoites [45], GAP50 is principally expressed in blood and gametocyte stages. In the blood, GAP50 is a constituent protein of the glideosome, an actin-myosin motor complex critical for erythrocyte invasion [56–58]. GAP50 expression continues into gametocytes [59], where its interaction with factor H [59] is being targeted as a candidate transmission blocking strategy [60]. At this time, it remains unknown whether a late-arresting/less-attenuated sporozoite would secondarily improve recall responses for GAP50 and other antigens tested in C57BL/6 mice.

The type of bio-informatic analysis conducted here has not been previously reported. Previous work has shown that rodent Plasmodia (*P. yoelii*/*P. berghei*) are evolutionarily much closer to one another than *P. falciparum*/*P. vivax* [61–65]. *P. yoelii*/*P. berghei* both arise from a rodent *Plasmodium* clade whereas *P. falciparum* is in the hominid clade and *P. vivax* in the monkey clade [66, 67]. Not surprisingly, there was greater amino acid homology of sufficient lengths to constitute potential class I MHC peptides (≥8 amino acids) for *P. yoelii*/*P. berghei* compared to *P. falciparum*/*P. vivax*. Nonetheless, the listings of shared syntenic peptides may help to identify protein targets that are in the right time and place to be T cell targets in heterologously immunized mice. These peptides also constitute one strategy for developing pan-*Plasmodium* T cell vaccines. Given the large evolutionary distance between *P. falciparum* and *P. vivax*, more closely related *P. falciparum* strains should also be evaluated. A number of these *P. falciparum* strains are now available for CHMI studies (e.g., *P. falciparum* 7G8 and *P. falciparum* NF135.C10) and will certainly share many more homologous syntenic peptides with *P. falciparum* than for the *P. falciparum*/*P. vivax* pair. Continued bio-informatics and experimental analysis of common peptides between species and strains may reveal novel strategies for developing malaria vaccines against one or more of the human-infecting strains.

One potential limitation of this study is that the infectivity of *P. yoelii* and *P. berghei* for mice differs. *P. berghei* infectivity is generally greater than *P. yoelii* in C57BL/6 mice whereas *P. yoelii* is more infectious in BALB/c mice (reviewed in [68]). However, the same number of parasites was administered regardless of species since there is no clear method for titrating the dose of one species against the other. The less infective species was used as the priming dose (*P. yoelii* in C57BL/6 mice or *P. berghei* in BALB/c mice). In addition, the same species was always used as the final dose, thereby minimizing the infectivity difference at the time of measurement. Nonetheless, infectivity differences may need to be accounted for in efforts to design optimally protective vaccination regimens. For instance, it may be desirable to use the most infectious species first to prime a larger number of responses or it may prove better to use the more infectious sporozoites secondarily to induce a larger recall response.

## Conclusion

In summary, these data categorize CD8^+^ T cell responses induced by *Plasmodium* sporozoites into (a) those primed but never recalled by sporozoite re-exposure; (b) primed and recalled by homologous sporozoites; or, (c) primed and recalled by heterologous but not homologous re-sporozoites. The next phase of this work is to determine how heterologous immunizations affect antibody responses and to determine if two-dose heterologous or combination dose immunizations can be designed to achieve sterile protection. A possible strategy is to prime mice or humans with sporozoites of a single species or strain and then boost mice with that same strain plus sporozoites from an additional species or a different strain. In addition, if low abundance and/or late liver stage antigens are found to be important through sporozoite- or subunit-based experimentation, sporozoite-based vaccines could potentially be empowered to expand such responses if recombinant parasites were engineered to express such live stage antigens earlier as pre-formed proteins in salivary gland sporozoites.

## Additional files

10.1186/s12936-016-1295-5 PyCSP-specific T cells expand while PyL3-specific T cells do not following multiple *P. yoelii* RAS immunizations BALB/cj mice were immunized one or three times with 1-2x10^4^ *P. yoelii* 17XNL RAS at 3-week intervals and monitored T cell responses by *ex vivo* IFNγ ELISPOT using H2-K^d^-binding peptides from PyCSP (**A**, SYVPSAEQI) and PyL3 (PY05881) (**B**, GYKSGMSHI). Bars display mean value and error bars show the 95% confidence interval; *p <0.05, **p <0.01, ***p <0.001, ****p <0.0001, Student’s t-test.
10.1186/s12936-016-1295-5 Title: Proteins subjected to bioinformatics analysis and listing of all syntenic orthologue pairs. This file provides multiple worksheets listing species-by-species protein identifiers (PlasmoDB gene ID) as well as worksheets listing the syntenic orthologue pairs for *P. yoelii* 17XNL/*P. berghei* ANKA and *P. falciparum* 3D7/*P. vivax* Sal1. These pairings were used as input pairings for Additional files 3 and 4.
10.1186/s12936-016-1295-5 Title: Common peptides from syntenic orthologues of *P. yoelii* 17XNL and *P. berghei* ANKA. This file provides multiple worksheets listing ≥8 amino acid contiguous stretches of homology for *P. yoelii* 17XNL/*P. berghei* ANKA syntenic orthologue pairs for all lifecycle stages, sporozoite stage and liver stage proteins.
10.1186/s12936-016-1295-5 Title: Common peptides from syntenic orthologues of *P. falciparum* 3D7/*P. vivax* Sal1. This file provides multiple worksheets listing ≥8 amino acid contiguous stretches of homology for *P. falciparum* 3D7/*P. vivax* Sal1 syntenic orthologue pairs for all lifecycle stages, sporozoite stage and liver stage proteins.
10.1186/s12936-016-1295-5 *P. berghei* and *P. yoelii* sporozoites trigger the same IFNγ-producing L3-specific T cell responses. (**A–B**) BALB/cj mice were immunized with 2.5x10^4^ WT *P. yoelii* 17XNL (black bars) or *P. berghei* ANKA (open bars) sporozoites under chloroquine treatment (0.8 mg chloroquine ip daily). Six days post-immunization, splenocytes were assessed for PyCSP- and L3-specific responses by IFNγ ELISPOT. (**A**) Examples of IFNγ spots for each condition and (**B**) Mean responses to PyCSP and PyL3 peptides for both *P. yoelii* and *P. berghei* species. PyCSP responses to *P. berghei* sporozoites versus *P. yoelii* sporozoites was significantly different (p <0.05 Student’s t-test). (**C**) BALB/cj mice were immunized with 1x10^4^ *P. berghei* ANKA RAS and splenocyte responses to the PyCSP (SYVPSAEQI) and PbCSP (SYIPSAEKI) epitopes were assessed six days post immunization by IFNγ ELISPOT. Error bars in B-C are 95% CI.
10.1186/s12936-016-1295-5 Near total loss of B220^+^ B cells from peripheral blood following anti-CD20 antibody treatments in BALB/cj mice. Percentage of B220^+^ B cells in peripheral blood following mock or anti-CD20 antibody treatment of mice undergoing sporozoite immunization. Anti-CD20 antibody treatment resulted in near total loss of B cells from the peripheral blood. *** p <0.001, Student’s t-test.
