# Supplementary figures and images for: Defining rules of CD8+ T cell expansion against pre-erythrocytic Plasmodium antigens in sporozoite-immunized mice
Source: Malar J. 2016 Apr 26;15:238. doi: 10.1186/s12936-016-1295-5 (PMC4845300; doi:10.1186/s12936-016-1295-5)

## Slide 1
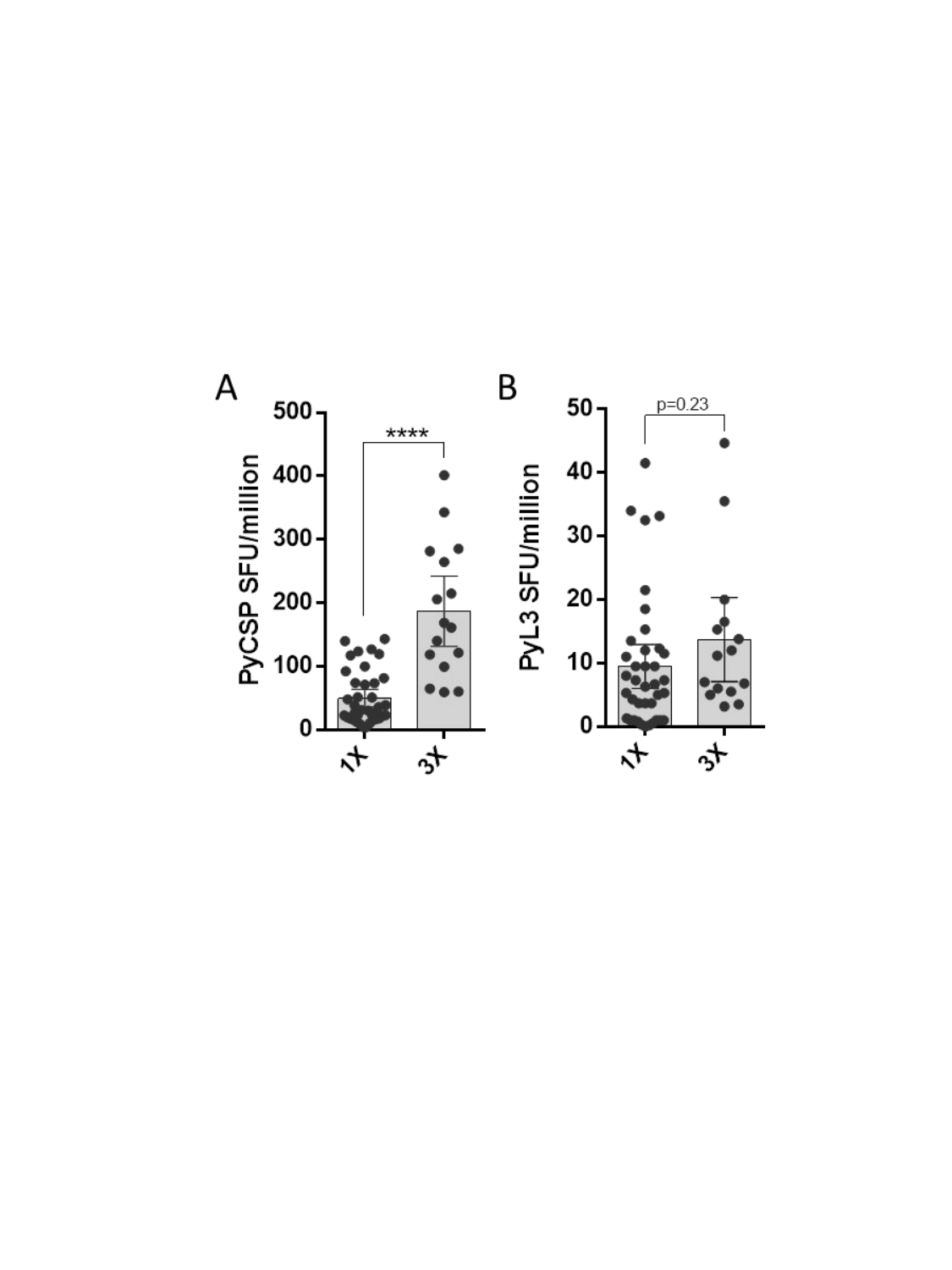

Supplement: Supplementary file 1 — 10.1186/s12936-016-1295-5 PyCSP-specific T cells expand while PyL3-specific T cells do not following multiple P. yoelii RAS immunizations BALB/cj mice were immunized one or three times with 1-2x10^4^ P. yoelii 17XNL RAS at 3-week intervals and monitored T cell responses by ex vivo IFNγ ELISPOT using H2-Kd-binding peptides from PyCSP (A, SYVPSAEQI) and PyL3 (PY05881) (B, GYKSGMSHI). Bars display mean value and error bars show the 95% confidence interval; *p <0.05, **p <0.01, ***p <0.001, ****p <0.0001, Student’s t-test. [file 12936_2016_1295_MOESM1_ESM.pptx]

## Slide 1
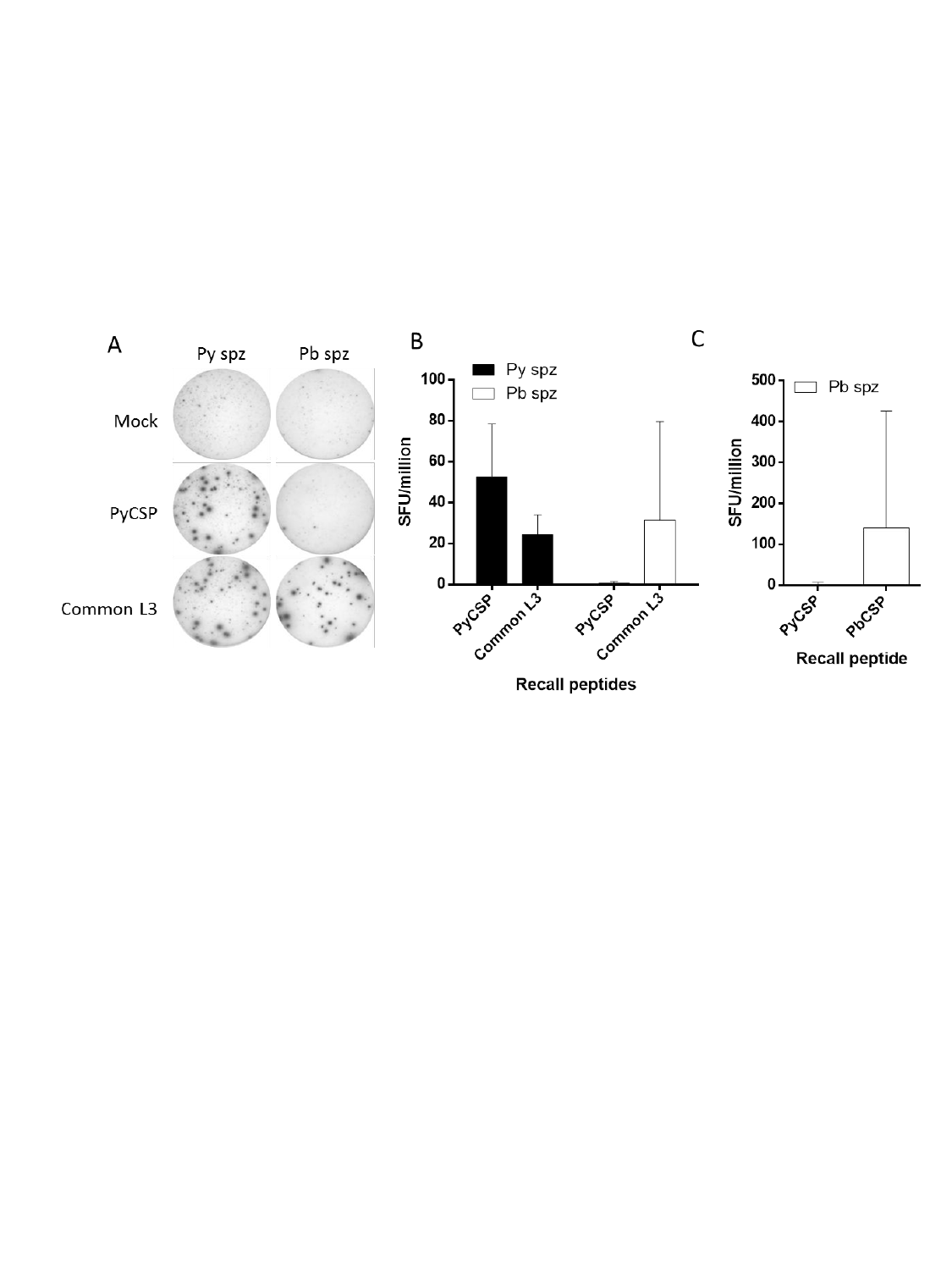

Supplement: Supplementary file 5 — 10.1186/s12936-016-1295-5 P. berghei and P. yoelii sporozoites trigger the same IFNγ-producing L3-specific T cell responses. (A–B) BALB/cj mice were immunized with 2.5x10^4^ WT P. yoelii 17XNL (black bars) or P. berghei ANKA (open bars) sporozoites under chloroquine treatment (0.8 mg chloroquine ip daily). Six days post-immunization, splenocytes were assessed for PyCSP- and L3-specific responses by IFNγ ELISPOT. (A) Examples of IFNγ spots for each condition and (B) Mean responses to PyCSP and PyL3 peptides for both P. yoelii and P. berghei species. PyCSP responses to P. berghei sporozoites versus P. yoelii sporozoites was significantly different (p <0.05 Student’s t-test). (C) BALB/cj mice were immunized with 1x10^4^ P. berghei ANKA RAS and splenocyte responses to the PyCSP (SYVPSAEQI) and PbCSP (SYIPSAEKI) epitopes were assessed six days post immunization by IFNγ ELISPOT. Error bars in B-C are 95% CI. [file 12936_2016_1295_MOESM5_ESM.pptx]

## Slide 1
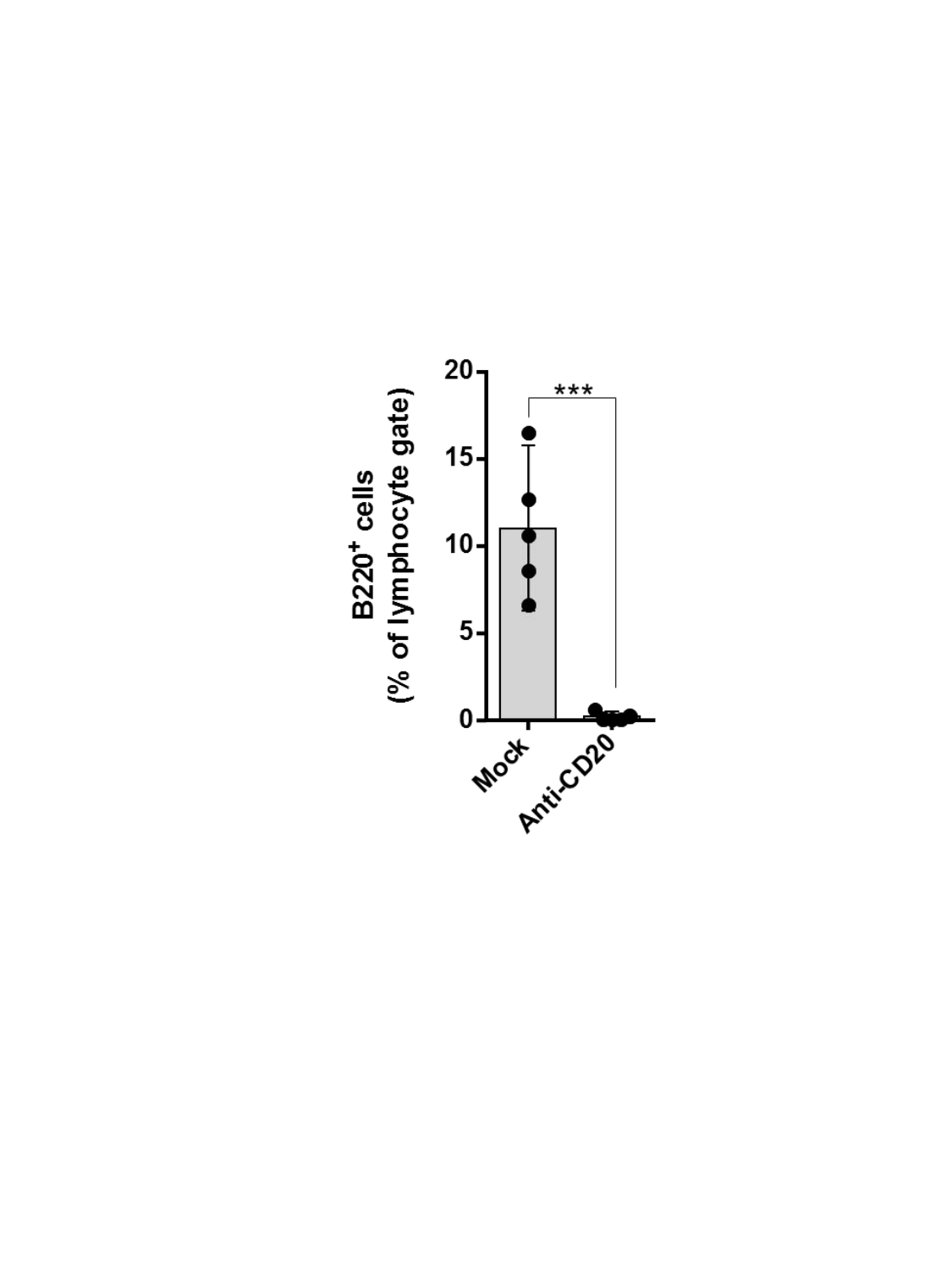

Supplement: Supplementary file 6 — 10.1186/s12936-016-1295-5 Near total loss of B220+ B cells from peripheral blood following anti-CD20 antibody treatments in BALB/cj mice. Percentage of B220+ B cells in peripheral blood following mock or anti-CD20 antibody treatment of mice undergoing sporozoite immunization. Anti-CD20 antibody treatment resulted in near total loss of B cells from the peripheral blood. *** p <0.001, Student’s t-test. [file 12936_2016_1295_MOESM6_ESM.pptx]
